# Role of bacteria in cancers and their therapeutic potential: Review of current knowledge

**DOI:** 10.22038/ijbms.2024.77667.16798

**Published:** 2025

**Authors:** Wojciech Wawrety, , Anna Kedziora

**Affiliations:** 1 Department of Microbiology Faculty of Biological Sciences University of Wroclaw Przybyszewskiego 63, 51-148 Wroclaw, Poland

**Keywords:** Bacteria, Immunotherapy, Microbiota, Neoplasms, Tumor microenvironment

## Abstract

Cancers are extremely dynamic diseases that can actively cause refractorines to be gained from applied therapies, which is why they are at the forefront of deaths worldwide. In this literature review, we covered the most recent and important discoveries regarding the influence of human microbiota, including tumor bacteriome, on the development and treatment of cancer. Advances in research on microbial communities have enabled us to discover the role of the human microbiome in the development and course of this disease, helping us understand neoplasms better and design new potential therapies. As we show through our findings, by immunomodulation and the secretion of certain chemical substances, the correct bacteriome of the intestinal tract, respiratory system, or skin can protect humans against cancer development and help during the treatment process. Bacteria also reside inside tumors, forming part of the tumor microenvironment (TME), where they interact with immunological and cancer cells in many complex ways. Some bacteria, such as *Pseudomonas aeruginosa* or *Akkermansia muciniphila*, can stimulate anticancer cell-mediated immune responses or even directly lead to cancer cell death. We also present the clinical possibilities of using some live, usually modified bacteria to develop bacteriotherapies. Modifying the gut microbiome to stimulate standard treatment is also important. Research on the microbiome and cancer remains a challenging topic in microbiology, having a great potential for advancements in cancer therapy in the future, and is continuously becoming a more and more popular field of research, as shown by our statistical analysis of PubMed data.

## Introduction

Cancer treatment is one of the greatest challenges in modern science and medicine. It is the leading or second leading cause of premature death in 112 countries worldwide. In 2020, 19.3 million new cases of cancer were recorded worldwide, of which female breast cancer (11.7%), lung cancer (11.4%), prostate cancer (7.3%), nonmelanoma skin cancer (6.2%), large intestine (6.0%), and stomach (5.6%) were the most common (1). However, the incidence of this disease has been steadily increasing since “the second epidemiological transition”; contrary to popular belief, malignant tumors are not a new problem. Numerous archaeological studies have shown the occurrence of bone metastases in Neolithic and ancient communities around the world, which may be related to exposure to natural carcinogens by members of these communities (2).

Since then, humanity has made major advances in understanding, diagnosing, and treating cancer. It is estimated that 3,495,700 deaths were avoided in the United States between 1992 and 2019 (3). However, despite these positive findings, the amount of data available remains alarming, and the scientific and medical community faces numerous problems when using both traditional and new treatments. These include diagnostic difficulties, temporal and spatial heterogeneity of tumors, resistance to treatment, presence of cancer stem cells, aggressive metastases, complications related to the tumor microenvironment, and toxicity of therapeutics against human cells (4).

The heterogeneity problems include genome instability, epigenetic modifications, and altered regulation of gene expression, which often make it impossible to treat cancer as a homogeneous disease (5). For example, the reduced chances of a positive response to treatment with trastuzumab and lapatinib are related to the heterogeneous distribution of Erb2 (HER2) receptors on the surface of gastrointestinal and breast cancer cells (6). Resistance to chemotherapy, immunotherapy, or targeted therapy also results from changes at the molecular level. For example, apolipoprotein B mRNA editing enzyme (APEC) and catalytic polypeptide (APEC) enzymes lead to genome and chromosome instability and chromotripsy, accelerating the clonal evolution of cancer. As a result of these and other events, defective cells acquire mutations in ESR1 or in the Pi3K pathway, which are associated with resistance to targeted therapy (7). One study using *Saccharomyces cerevisiae* as a model organism showed that pdr3Δ strains were resistant to cytotoxic cantharidine. Moreover, its presence in the *in vitro* environment caused overexpression of the *PDR1 *and *PDR5* genes in yeast, indicating the role of ABC transporters in cell defense against the effects of chemotherapeutic agents and the risk of developing resistance by tumors (8). These transporters are especially abundant in cancer stem cells (CSCs). This subpopulation of cells is up to 50 times more likely to cause a new neoplastic disease. It is an overwhelming problem, often leading to recurrence after the end of therapy that is not fully effective (9). Recently, increasing attention has been given to the role of the tumor microenvironment (TME) and the interactions between cancer cells, immune cells, and bacteria in the TME. Tumors modify their microenvironment to evade the cellular response of the human immune system and facilitate further invasion. For example, tumor-associated macrophages (TAMs) activate the JAK2/STAT3 pathway by secreting IL-6, which leads to aggressive metastasis of colorectal cancers (10).

These problems have recently prompted scientists to look for therapeutic solutions that can overcome the difficulties associated with getting the drug to the deepest parts of the tumor and that can work as effectively as possible, regardless of the heterogeneity and molecular changes in cancer cells. Microbiome modifications and bacteriotherapy, which can directly modulate the TME, are believed to have enormous clinical potential. A noticeable trend related to the study of the human microbiome is associated with groundbreaking discoveries linking the participation of various species, especially intestinal bacteria, in the formation of numerous diseases, including neurodegenerative diseases (NDs), IBS, IBD, autism, depression, and finally cancer (11, 12). In this article, we would like to draw attention to the role of the human bacteriome, including the bacterial microbiome inside cancerous tumors, in the development and progression of cancerous diseases and to indicate the current attempts and possibilities for developing cancer bacteriotherapy in the future.

### Review method

Original studies and review papers regarding the role of the human microbiome, bacteria in the tumor microenvironment, and bacteriotherapy were searched for using commonly used databases, such as NCBI PubMed and Google Scholar with keywords such as ‘bacteria in tumor microenvironment,’ ‘*Listeria monocytogenes* in cancer treatment,’ etc. The credibility of literature sources was checked with MyBib online tool. Only the newest studies from reliable sources were included in the review. 

### Statistical analysis

To assess the supposedly rising interest in the topic covered in this paper, results by year for „bacteria and cancer” keyword searches were obtained from the NCBI PubMed database (accessed on 11/03/2024). Linearised non-linear regression analysis between the consecutive years (1914-2023) and results by year was performed using StatSoft Statistica 13.3.721.1 software. The analysis was performed with a significance level of α=0.05.

### Human microbiome and its role in cancer

The human body is inhabited by over 100 trillion microorganisms (13), including bacteria, viruses, fungi, and archaea. Among the bacteria, the *Firmicutes*, *Bacteroidetes,* and *Actinobacteria* are the most common in individual parts of the human body and inhabit the oral cavity, skin, respiratory tract, intestines, and genitourinary system. *Fusobacteria* and *Proteobacteria* also play important roles (14). Both the individual species and the total normal composition of the human microbiota are involved in the suppression of carcinogenesis and the positive response to applied therapies (Table 1). The role of bacteria in cancer diseases is related to, among other factors, their competition with “carcinogenic” bacteria for nutrients, secretion of anticancer metabolites, and interaction of these bacteria with receptors on the surface of cancer cells or the immune system (immunomodulation)(15).

The impact of the intestinal microbiota composition on various health statuses and diseases has been the subject of intensive research. Recent comparative statistical and metagenomic analyses have shown that appendectomy, which is believed to be a large reservoir of gut bacteria, increases the risk of developing colorectal cancer (CRC) by as much as 73% (*P*<0.001) through significant changes (*P*=0.038) in the species composition of the gut microbiome. *Bacteroides vulgatus*, *Bacteroides fragilis*, *Veillonella dispar*, *Prevotella ruminicola*, *Prevotella fusca*, *Prevotella dentalis,* and *Prevotella denticola* have been found to promote CRC development, while *Blautia* sp. SC05B48, *Colinsella aerofaciens*, *Lachnospiraceae* *bacterium* Choco 86, *Enterococcus hirae,* and *Blautia* sp. YL58 has been found to play a protective role (16).

The development of CRC and gastric cancer (GC) is also associated with changes in oral bacteria. A decrease in the diversity and abundance of bacterial species in oral samples was observed in individuals with GC and CRC, as indicated by a reduction of the Shannon index (*P*=0.05). These phenomena were accompanied by a percentage increase in the share of *Firmicutes* (especially *Streptococcus*) and *Herbaspirillum* and a decrease in the abundance of *Proteobacteria*, especially *Haemophilus* and *Neisseria* (*P*=0.05, *P*=0.01, and *P*=0.001)(17). In the case of CRC, the proportion of *Fusobacteria* in oral samples decreases (*P*=0.001) (with a simultaneous increase in stool samples). The representative *Fusobacterium nucleatum*, according to the generally accepted consensus, can migrate from the oral cavity to the intestines, where it promotes the proliferation and chemoresistance of various types of cancer (17, 25).

The skin microbiota also plays an often underestimated role. In patients with cutaneous lymphoma who underwent nbUVB phototherapy, increased survival and a positive response to treatment were shown to be associated with greater α diversity of bacteria on the skin surface and a greater proportion of *Staphylococcus* species (especially *S. capitis*, *S. epidermidis,* and *S. warneri*), *Acinetobacter* and *Anaerococcus* (q<0.05)(19). Some coagulase-negative staphylococci can limit the growth of *S. aureus*, which is the source of many harmful and carcinogenic compounds, such as hemolysins, serine proteases, and phenol-soluble modulins, which can lead to the formation of skin cancers by stimulating inflammation (18, 19).

Numerous mechanisms are involved in the differences in the microbiome composition of sick and healthy people and those varying in their responses to therapies. As already mentioned, some bacteria produce numerous anticancer compounds and metabolites *in vivo*. Nasopharyngeal cancer (NPC) is also associated with changes in the local microbiota. Bacteria of the genus *Granulicatella* are most likely involved in adverse changes in the oral microbiota and the formation of carcinogenic nitrosamines in patients. In contrast, nasopharyngeal biopsy samples showed a significant decrease in the number of *Pseudomonas* and *Acinetobacter* bacteria **(***P*=0.0**01)**. In particular, *Pseudomonas aeruginosa* is a source of metabolites with anticancer activity, such as exotoxin A, exopolysaccharides, and L-asparaginase (20).

Analyses of samples taken from patients with non-small cell lung cancer (NSCLC) treated with a PD-1 inhibitor and healthy individuals showed that *Akkermansia* *muciniphila*, *Rikenellaceae*, *Bacteroides*, *Peptostreptococcaceae*, *Mogibacteriaceae,* and *Clostridiaceae* had smaller shares of the gut microbiome (*P*≤0.05). *A. *muciniphila participates in maintaining mucosal integrity by degrading mucins and preventing inflammation, while *Bacteroides* spp. and *Clostridium* spp. metabolize indole acetic acid to 3-methylindole (skatole), a compound with anticancer properties. Moreover, the presence of short-chain fatty acids (SCFAs) produced by these bacteria was associated with a positive response to treatment (21).

SCFAs, products of bacterial fermentation of fiber, can directly bind to FFAR2, FFAR3 *(*a fatty acid receptor), and G protein-coupled receptors (GPCRs) located on the surface of human large intestine cells. FFAR2 stimulation prevents the migration of neutrophils and the increase in the secretion of proinflammatory cytokines (TNFα and IL-17), promotes the proliferation of regulatory T cells, prevents excessive inflammation, and favors the growth of *Bifidobacteria* in the intestinal lumen. In turn, the activation of HCAR2 by butyric acid stimulates the secretion of the anti-inflammatory cytokine IL-18, inhibits proliferation, and leads to the apoptosis of CRC cells (22). The roles of SCFAs produced by *Porphyromonas gingivalis* and *F. nucleatum* in the oral cavity seem different. According to *in vitro* studies, stimulating some human ameloblastoma cell lines with sodium butyrate increases the expression of EGF and TGFβ1 mRNA, which are involved in the proliferation and migration of cancer cells. Together, these cytokines may up-regulate the expression of laminin LMβ3, which is involved in the migration of cancer cells. These findings suggest that SCFAs in the oral cavity indirectly contribute to the increase in invasion and metastasis of cancers, in contrast to the beneficial effects of these acids in the intestines (26).

Lipopolysaccharide (LPS) from gram-negative bacteria is known to promote inflammation, toxic shock, carcinogenesis, and tumor metastasis. However, when the apoptosis receptor antagonist (IAP) inhibitor SM-164 was combined with LPS, an increase in the activation of apoptosis in the MDA-MB-231 triple-negative breast cancer (TNBC) cell line was observed. In a mouse model, LPS also led to the regression of tumors overexpressing IAP1/2 ER+ in combination with an IAP antagonist by inducing apoptosis through the secretion of TNFα by activating TLR4 and its adapter protein MyD88 (27).

The microbiome is also involved in direct immunomodulation. The activity of the immune system is directly related to the course of neoplastic diseases. Some bacteria, such as *Bifidobacterium pseudolongum*, *A. municiphila,* or *Lactobacillus johnsonii*, secrete inosine and hypoxanthine, which, by binding to A2A adenosine receptors (A2AR), leads to the proliferation of cytotoxic T cells and a more effective response to therapy with checkpoint inhibitors (ICIs) (23). Some bacteria, such as the probiotic strain *Lactobacillus rhamnosus*, can produce extracellular vesicles (EVs) that have direct anticancer effects on liver cancer cells. However, the related *L. johnsonii* abolishes genotoxicity in mouse models of ataxia-telangiectasia, preventing the development of leukemia (24).

Immunomodulation in combination with ICIs is highly important for determining the response to immunotherapy. For example, in melanoma patients treated with ICIs, *Gemmiger formicilis*, *Dorea formicigenerans*, *Ruminococcus bromii*, *Clostridia*, *Bacteroides thetaiotaomicron*, *Holdemania filiformis*, *Bifidobacterium longum*, and *Colinsella earofaciens* were shown to be associated with a positive response to treatment, which was correlated with CD4+ Th1 cell polarization, which is involved in the cellular anticancer response (28).

### Role of the bacteriome in the tumor microenvironment

**Figure 1:**
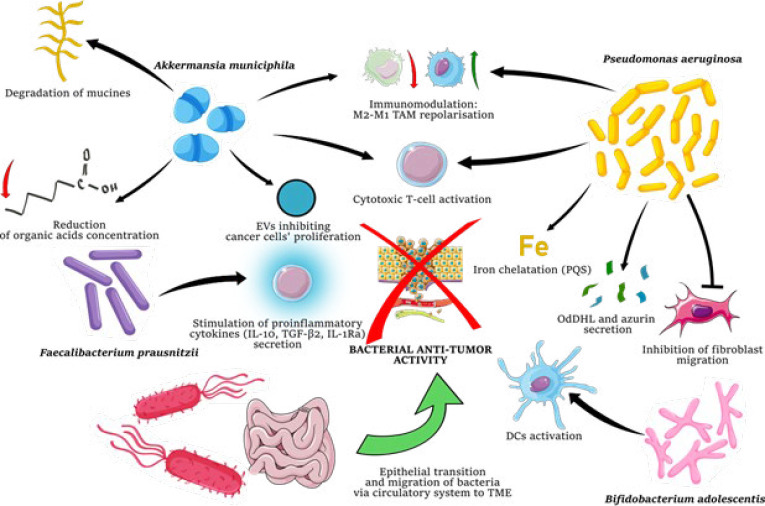
Examples of bacterial antitumour activity within the TME (*Akkermansia municiphila, Pseudomonas aeruginosa, Bifidobacterium adolescentis, Faecalibacterium prausnitzii*)

In recent years, we have become increasingly convinced that some parts of the human body previously considered sterile are inhabited by microbiota. Intriguingly, numerous studies have shown the presence of bacteria even inside cancerous tumors. Because the bacteria present in tumors are part of the TME, they are sometimes referred to as the tumor microbe microenvironment (TMME). The TMME is usually composed mainly of *Proteobacteria* and *Firmicutes*, similar to the gut microbiome. By interacting with human cell receptors, secreting compounds, and metabolites, or directly engaging in oncolysis, TMME bacteria play a significant role in immunomodulation, response to treatment, and ultimately survival (29, 30)(Figure 1). The composition of the TMME varies among cancer types and subtypes. For example, the shares of the genera *Alkanindiges* (*P*=0.012), *Micrococcus* (*P*=0.019), *Caulobacter* (*P*=0.011), *Proteus*, *Brevibacillus*, *Kocuria* (*P*=0.019*),* and *Parasediminibacterium* are greater in estrogen receptor-negative (ER-) than in estrogen receptor-positive (ER+) breast cancers (31).

According to the driver-passenger model, TMME bacteria can be divided into those that occur in a given place in the body even before the appearance of cancer and may play a role in its formation, as well as those that colonize an already-formed tumor. The most well-known examples are bacteria that can lead to cancer formation. For example, studies have shown that a dysregulated local lung microbiota can cause cancer cell proliferation and excessive neutrophil activity, e.g., by stimulating γδ T lymphocytes to secrete proinflammatory factors and overexpressing PD-1 and CD103 surface receptors (32). Gallbladder colonizing *Salmonella enterica* subsp. *enterica* sv. Typhi produces a CDT toxin that causes double-strand breaks in DNA. After internalization by bacteria inside the cell, the tripartite CdtB-PltA-PltB complex can be secreted into the intercellular space by vesicular transport, where it can lead to DNA damage in neighboring cells and promote carcinogenesis by activating the ataxia-telangiectasia mutated signaling network (ATM)(33). However, the above-cited *F. nucleatum* strains may serve as negative examples of intracancer “passenger” bacteria. Significant increases (*P*=0.05, *P*=0.01) in proinflammatory cytokines (IFN-γ, TNF-α, IL-6, IL-12, IL-17A, IL-9, CXCL1, MCP-1, and eotaxins) increase the level of MDSC suppressor cell infiltration, decrease the level of natural killer cells and T cells (CD3+, CD4+, CD8+), disturb the structure of the intestinal bacteria, and consequently increase the number of liver metastases compared to those in the control group (*P*=0.01). This bacterium is present inside metastatic tumors in the liver (34).

Although the literature focuses mainly on the role of bacteria in inducing mutations, promoting metastasis, and promoting tumor aggressiveness, specific bacterial species have opposite effects, and understanding their role is essential in developing new therapies. According to one study, the presence of *Pseudoxanthomonas, Saccharopolyspora, *and *Streptomyces* sp. in the TME and the species diversity of the TMME were associated with increased survival of patients with pancreatic cancer (35).

Cancer-colonizing bacteria can probably penetrate the intestinal epithelium by regulating its permeability, and subsequently, through the circulatory system, they are able to reach cancerous tissues. One of the pioneering studies using microscopy and metabolomics techniques, AFADESI-MSI, showed that oral administration of *A. muciniphila* to mice was able to modulate metabolic pathways in lung cancer. This bacterium reduced the concentration of lactic, glutamic, succinic, and malic acids, key products of abnormal metabolism in cancer cells. The nucleotide biosynthesis pathway was also disrupted (reduced concentrations of AMP, ADP, GMP, UMP, and uric acid), which is necessary for tumor progression. Moreover, the temporary presence of *A. muciniphila* in the blood and an increase in the share of bacteria from the genera *Asinibacterium*, *Lactobacillus*, *Bacteroides*, *Dubosiella*, *Methylovirgula*, *Romboutsia,* and *Bosea* in the TMME were observed compared to those in the control group (*P*=0.05)(36).

The affinity of *A. muciniphila* for the TME is most likely due to anaerobic conditions (this organism is an obligate anaerobe) and the high concentration of nutrients in the tumor. Interestingly, metastasis-promoting mucins are overexpressed in CRC and are a source of carbon, nitrogen, and energy (37). Notably, 60% of the extracellular vesicles (EVs) secreted by this bacterium (strain ATCC BAA-835) inhibited tumor growth in a mouse model of prostate cancer (RM-1) on day 13 of tumor growth. Additional *in vivo *and *in vitro* studies have shown that the beneficial effects of *A. muciniphila* EVs are associated with the positive regulation of CD8+ GZMB+ and IFN-γ+ T-cell infiltration into tumors, the promotion of a phenotypic shift toward beneficial M1 macrophages, and the increased ability of these cells to inhibit the proliferation and invasion of cancer cells (38).

The common pathogenic rod-shaped bacterial strain *P. aeruginosa *has unusual properties and therapeutic potential within the TMME. Cystic fibrosis (CF) patients whose respiratory tracts are commonly colonized with this bacterium have significantly lower rates of melanoma and breast cancer. In the plasma of patients with CF, the levels of azurin, a pigment secreted by *Pseudomonas*, are significantly elevated (*P*=0.0001)(39). *In vitro* studies have shown that *azu* gene expression and the consequent secretion of azurin in *P. aeruginosa* isolated from CF patients are increased in the presence of breast cancer cells (MDA-MB-231) and melanoma cells (Mel-2). Azurin secretion was shown to be associated with the exposure of bacteria to aldolase A secreted by tumor cells in the presence of *P. aeruginosa*, which, together with MUC-1 mucin, determines the adherence of *P. aeruginosa* to tumor cells. Microscopy visualization (TEM) also showed the presence of *P. aeruginosa* inside the tumor cells. Notably, in patient samples, the *azu* gene was detected in more primary tumors than in metastatic tissues (39). This study demonstrated the remarkable interactions between tumors and bacterial cells and the relationship between tumor invasiveness and azurin secretion by *P. aeruginosa* in the TMME. Notably, through the secretion of azurin and the P28 peptide, *P. aeruginosa* is also able to inhibit the migration of fibroblasts induced by VEGF and FGF (40).

Another study using a clinical isolate of *P. aeruginosa* performed in a mouse model of lung epithelial carcinoma showed a significant decrease in the growth of tumors in mice infected with *P. aeruginosa* compared to those in the control group. In addition, infected mice exhibited long-term antitumor immunity. This process involved the ability of this bacterium to promote necroptosis by activating the TLR4 receptor, stimulating DCs to secrete specific cytokines (Il-1β, IL-6, MCP-1, and TNF-α), increasing the share of CD4+ and CD8+ T cells, and decreasing the share of suppressor cells such as MDSCs and M2 within the TME (45).

Furthermore, factors secreted by *P. aeruginosa* have synergistic effects on doxorubicin. Transcriptomic studies have shown that this anticancer drug induces the production of PQS *(Pseudomonas quinolone signal)*, which can chelate the iron needed for the proliferation of cancer cells and increase the anticancer activity of doxorubicin (41). In addition, the autoinducer of the quorum sensing (QS) system of *P. aeruginosa,* N-3(oxododecanyl)-L-homoserine lactone (OdDHL), which is involved in the formation of biofilms by this bacterium, reduces the proliferation of breast cancer cells (MDA-MB-231) under various *in vitro* conditions and leads to their necrosis (42). These and other data highlight multiple mechanisms of *P. aeruginosa’s* anticancer activity and its secreted metabolites. This bacterium and the compounds secreted by it have powerful therapeutic potential.

Many other bacteria also play a significant role within the TME. The obligatory anaerobe *Bifidobacterium* sp. can accumulate inside tumors where it increases the effectiveness of anti-CD47 immunotherapy by interacting with dendritic cells and the cGAS-STING (cyclic GMP-AMP synthase - stimulator of interferon genes) pathway (43). The results of one study showed that exposure of CRC cells to *F. nucleatum* also leads to activation of the STING pathway through positive regulation of cGAS and STING phosphorylation *in vitro*. This bacterium can also reduce the proliferation of CRC cells in organoid models via the conjugated use of PD-L1 blockade by increasing the proportion of CD8+ TILs and IFN-γ+ CD8+ lymphocytes and activating the STING pathway. In turn, the probiotic bacterium *Bifidobacterium adolescentis* directly stimulates DCs and enhances the effectiveness of anti-PD-1 treatment (46). This finding indicates the complexity of the mutual impacts and impact networks within the TME. However, the bacterium *Faecalibacterium prausnitzii*, which belongs to the *Clostridia* class, positively regulates the expression of anti-inflammatory cytokines (IL-10, TGF-β2 and IL-1Ra) and has the opposite effect on proinflammatory cytokines (IL-6, TNF-α, and TNF-β) in lung cancer (44). Notably, *F. prausnitzii* is a bacterium whose participation in the TMME of lung cancer is significantly reduced (*P*=0.0004) (47).

### Future perspectives: Microbiome modifications and bacteriotherapy

The unusual properties of bacteria have prompted scientists to try to apply their potential in cancer treatment. Promising strategies include microbiome modifications, such as probiotic therapy and fecal matter transplants (FMTs); the possibility of using bacterial metabolites with anticancer properties; bacteriotherapy (BT) using live, often genetically modified microorganisms; and therapies using methods of synthetic biology.

According to the FAO/WHO definition, probiotics are “live microorganisms which, when administered in adequate amounts, confer a health benefit on the host” (48). Probiotics provide measurable results in the prevention, treatment, and alleviation of side effects associated with standard therapies. Probiotic strains are capable of inducing apoptosis and autophagy in cancer cells, reducing the expression of oncogenes, inhibiting the activity of kinases, reactivating tumor suppressor genes and preventing metastases. For example, *B. longum* stimulates the expression of suppressor miRNAs (miR-145 and miR-15) in mouse CRC (49). However, *L. acidophilus* and *L. rhamnosus* GG reduce the expression of CD147 glycoproteins on differentiated monocytes. These receptors, which are overexpressed in cancer cells, are involved in metastasis by stimulating angiogenesis and the expression of genes encoding metalloproteinases (50). The possibility of using probiotic anaerobic bacteria, such as *Clostridium butyricum* MIYAIRI 588, seems interesting as well. In clinical trials, the administration of this probiotic strain in combination with immunotherapy to people with non-small cell lung cancer significantly prolonged progression-free survival (PFS) (*P*=0.009). Positive results in the modulation of intestinal bacteria were visible 14-21 days after the start of probiotic therapy (51).

Many of the anticancer properties of lactic acid bacteria (LAB) arise from the properties of their primary metabolites, which are peptide bacteriocins. Nisin (a class I bacteriocin) synthesized by *Lactococcus lactis* or the pediocin *Pediococcus acidilactici* K2a2-3 (class II) has cytotoxic effects on some cancer lines and can be used as a drug to increase the effectiveness of standard chemotherapy (58, 59). *Streptomycetes actinomycetes* isolated from the environment are also a source of anticancer compounds. Salinisporamide A, an active metabolite of *Salinispora tropica* with cytotoxic properties, and diazepinomycin, which are produced by some strains of *Micromonospora* sp. and are capable of inducing apoptosis, can also be mentioned here (60).

Fecal transplants are a promising alternative to classical probiotic therapy. Many clinical trials are currently being conducted to test the effectiveness of FMT in cancer patients. FMT offers a promising opportunity to increase the effectiveness of ICIs in the future (61). Clinical studies have proven the efficacy of FMT in combating and preventing complications associated with anticancer therapies, such as *Clostridium difficile* infections and graft-versus-cell reactions, in the treatment of human leukemia (62).

For centuries, it has been well known that acute bacterial infections, such as erysipelas caused by *Streptococcus pyogenes* or gangrene caused by *Clostridium perfringens*, can lead to complete spontaneous regression of tumors. In the XIX century, Coley’s toxin (CT), i.e., the thermally inactivated bacteria *S. pyogenes* and *Serratia marcescens*, was successfully employed in treatment (54, 63). Although the advent of chemo- and radiotherapy ultimately led to the use of this pioneering method for the development of immunotherapy and bacteriotherapy, some studies indicate the possibility of reversing the problems associated with the use of these and other related methods. According to *in vitro* studies, CT significantly reduces the growth of certain cancer cell lines. CT regulates the expression of the cell cycle-regulating gene p21^waf^, induces apoptosis in cancer cells (activation of caspases and DNA fragmentation), stimulates the expression of the TLR2, TLR5, and TLR9 receptors in immune cells, and increases the effectiveness of cytotoxic T cells (54).

Some wild-type strains of bacteria show a specific tropism toward neoplastic tumors. *Salmonella Typhimurium*, *L. monocytogenes*, *Clostridium novyi *(-NT), *Clostridium butyricum,* and some strains of *Bifidobacterium* sp. or *E. coli* after intravenous injection are located in the TME and are capable of significant immunomodulation and cytotoxicity against cancer cells (63, 64). Understanding the mechanisms of colonization is highly important for studying the natural chemoattractance of strains against specific cell lines. In 2018, a team of scientists developed a microchip to study this phenomenon. It was shown that, compared with healthy cells, *E. coli* O157 cells exhibited a significant preference for chemotaxis toward microchambers containing lung cancer cells (NCI-H460)(65).

*Clostridium novyi*-NT is an obligate anaerobic, spore-forming, environmental or animal microbiome bacterial species belonging to the phylum *Firmicutes* that is devoid of the main virulence factor in the form of a phage encoding the α-toxin. *C. novyi*-NT spores selectively colonize hypoxic tumor regions, cause direct cytotoxicity due to the secretion of specific lipases, and activate the inflammatory response and cytotoxic T-cell activity while overcoming immunosuppression (55).

**Figure 2:**
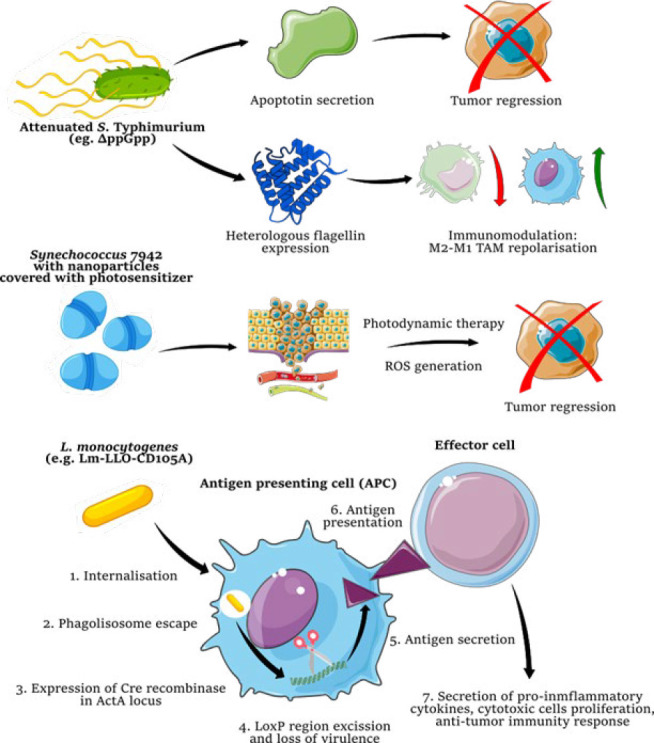
Examples of bacteriotherapy strategies

Another widely used experimental bacterium is *Salmonella enterica* subsp. *enterica* sv. Typhimurium strains from the *Enterobacteriaceae* family can grow under both aerobic and anaerobic conditions. Among the modified strains developed in the laboratory and tested in the research, the following should be mentioned: VNP20009, AR-1, ΔppGpp, SB842, MvP728, SL, and YB1. Despite its natural toxicity and the ability to effectively colonize cancerous tumors and inhibit the growth of numerous tumors in mice, *S.* *Typhimurium* did not yield the expected results as a monotherapy in phase I clinical trials (56, 57). Therefore, in addition to attempting to use “mixed” therapies, scientists are developing strains of *S. Typhimurium* with additional cytotoxic or immunomodulatory abilities. For example, the attenuated strain of *S*. *Typhimurium* was infected with the pCDNA3.1 plasmid, which contains the gene encoding apoptin, a protein that leads to tumor cell apoptosis via a p53-independent pathway. Similarly, in another study, the *S. Typhimurium* ΔppGpp strain was modified to express the heterologous flagellin *Vibrio vulnificus*, which was able to stimulate an antitumor response and macrophage repolarization from M2 to M1 in the mouse tumor TME (66)(Figure 2).

The possibility of using the intracellular bacterium* L. monocytogenes*, which belongs to *Firmicutes* (*Bacillota*), is also widely discussed. Its use in anticancer therapies is highly attractive. Owing to specific internalins, they can infect some immune cells (and move inside macrophages to various parts of the body, including cancerous tumors), producing the cytotoxic exotoxin listeriolysin O and phospholipase C. Additionally, as part of this modification, it is possible to use the ActA system, which enables this bacterium to polymerize eukaryotic actin and escape from infected cells (68). In addition, EVs secreted by DCs infected by this bacterium (provided that they are capable of secreting listeriolysin O) activate the innate antiviral immune response, which is associated with, among other factors, a significant increase in proinflammatory cytokines such as IL-1b and IL-12 p40 (69). One of the approaches used to address this issue is cancer vaccine development. *L. monocytogenes* can be engineered to “present” a fragment of the CD105 antigen (strain Lm-LLO-CD105A), which is overexpressed in rapidly proliferating epithelial cells. After infecting an antigen-presenting cell (APC) and escaping from the phagolysosome, Lm-LLO-CD105A delivers the antigen, enabling an effective antitumor response in the immune system (Figure 2). A renal tumor (RCC) study of this vaccine showed that *in vivo* administration of this vaccine to mice resulted in a significant reduction in tumor growth and progression, a reduction in the vascular network within tumors, increased infiltration of CD8+ and CD4+ T cells into the TME and expression of beneficial cytokines (IFN-γ, IL-2, TNF-α) and a reduction in the population of suppressor cells (Treg Cd4+Fox3+), confirming the effectiveness of the method (67).

In addition, there is the possibility of advanced attenuation of bacteria. Insertion of Cre recombinase into the actA locus results in its synthesis after infection of the cell by the bacterium. The Cre recombinase subsequently cleaves the loxP regions responsible for virulence while retaining its ability to express antigens as a vaccine. *Listeria* strains located within the TME can also be marked with radioactive ^188^Re radionuclides, enabling the selective cytotoxicity of radiotherapy toward tumors (52). Some bacteria, such as *Escherichia* and *Shigella*, can also be used as vectors for gene therapy. Bacterial vectors are more adaptable and easier to control than viral or liposomal vectors, offering more possibilities for numerous modifications. These strains may also carry plasmids or chromosomes with therapeutic genes or shRNA cassettes. Current research provides a good chance of using this method in the future (70).

Progress in science has given researchers an ever-wider range of microbiological, biotechnological, nanotechnological, and biophysical tools, through which it is possible to develop therapies that until now could be considered pure science fiction. A team of synthetic biologists recently obtained the so-called bacterial minicells from *E. coli*, which have acquired specific chemotactic abilities owing to the content of genes encoding flagellin. They can be used as carriers of various drugs (71). There is also the possibility of using bacteria in photodynamic therapy (PDT) for cancer. One study developed the phototrophic bacterium *Synechococcus* 7942 as a PDT adjuvant. *Synechococcus* was covered with nanoparticles combined with a photosensitizer that increased the effectiveness of PDT, while the ability to generate oxygen *in situ* allowed the infection to combat hypoxia and generate ROS, which is toxic to cancer cells (Figure 2). It is also interesting to cover *L. monocytogenes* with the cell membrane of erythrocytes (Lmo@RBC), which allows us to avoid a potentially harmful immune response to bacteria present in the blood (53).

### Rising interest in bacteria and cancer relations is statistically significant

**Figure 3:**
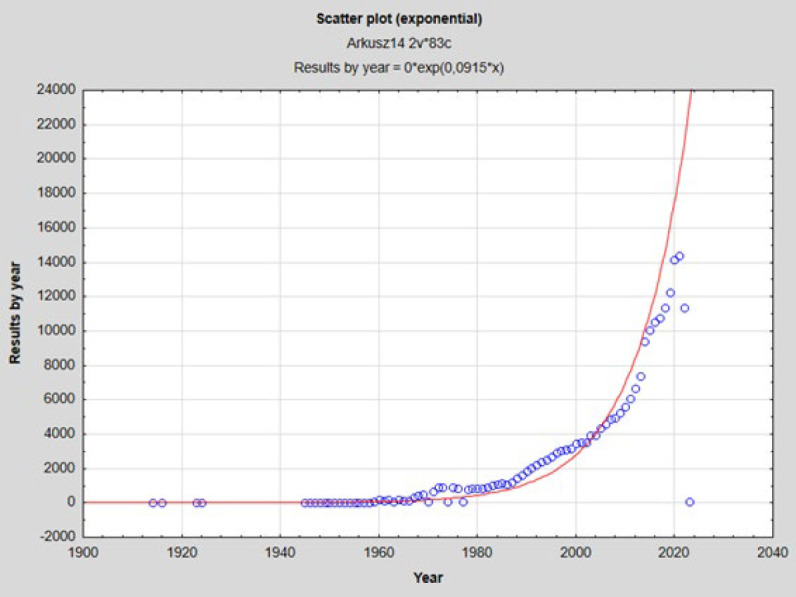
Exponential scatter plot representing rising interest in the microbiome-cancer relations’ research according to results by year of ‘bacteria and cancer’ on Pubmed between 1979 and 2022 (Source: PubMed, 11/03/2024)

As shown in the scatter plot (Figure 3.), the interest in bacteria and cancer relations on the PubMed database has been rising exponentially since the middle of the XX century. As shown by the results of linearised non-linear regression analysis, the correlation between consecutive years (1914-2023) and results by year is very high (R=0,93542036) and statistically significant (*P*=0.00 in F statistics). The distribution of remainders was close to normal. High determination coefficient (R^2^=0,87501126) points to the fact that the constructed regression model is mostly influenced by time, and we can expect even more interest and studies on the topic in the near future.

## Discussion

Cancer is currently one of the most deadly diseases of civilization, affecting millions of people every year and having a traumatic impact on the psyche of the patients themselves and their relatives. In the mid-twentieth century, after the discovery of radiotherapy and chemotherapy and due to advances in surgery, it was believed that these new methods would ultimately help win the war against cancer. Unfortunately, this did not happen, and at the same time, earlier “microbial” attempts to use bacteria as anticancer therapies were abandoned. The enormous advances made in recent years in the study of the microbiome and individual bacteria have allowed us to return to the original assumptions.

As can be seen in various studies, the composition of intestinal, skin, and lung microbiota differs significantly between healthy and cancer patients, influencing tumor development and survival. As we discovered during our review, microbial metabolites, such as SCFAs, LPS, inosine, and hypoxanthine, influence the expression and activity of various cell receptors, e.g., FFAR2, FFAR3, TLR4, or A2R. Bacteriome within the tumor microenvironment significantly interfers with cancer and immune cells, resulting in various responces to treatment and disease progression. *Akkermansia municiphila, P. aeruginosa, *and *Bifidobacterium *sp. are examples of TME bacteria having anti-cancer characteristics, being able to regulate metabolic pathways, increace anticancer drugs’ activity, modulate immune cells’ activity or having direct anti-proliferative effects, among others. Various innovative ideas for bacteriotherapy benefit from rapid advances in genetic engineering and synthetic biology to modify bactetia for, e.g., heterologous proteins’ expression, reduction of virulence, or as drug or nanoparticle vectors.

Unfortunately, the usage of bacteriotherapy remains highly limited and challenging. The only approved bacteria-based therapy, the BCG vaccine, is ineffective in 30-50% of non-muscle-invasive bladder cancers and 5% of patients develop severe side effects, such as sepsis. Among other significant problems are incomplete lysis of tumors and problems with targetting some metastatic tissues (72). Furthermore, bacterial compounds, such as LPS (lipopolysaccharide), CpG islands, or lipopeptides, are capable of initiating immunological response leading to immunosuppression or even, in the case of LPS, prompting *KRAS* mutations through unfavorable inflammation within the TME (73). As can be seen, the equilibrium between attenuation needed to evade excessive immunological response and saving the most important virulence factors indispensable for effective anti-cancer effects is highly problematic (74).

Microbiome research provides possibilities for discovering complex interactions that may prevent or promote cancer. In recent years, there has been a noticeable increase in interest in this topic (Figure 3). However, a large part of the related research is circumstantial; we observe variable compositions of intestinal bacteria in different physiological states, but explaining the causes of these differences often remains impossible. Accurate knowledge of the biology of interactions between individual species of bacteria; proteomic, metabolomic, and network analyses; and methods of classical microbiology must be conducted with appropriate regularity to fully understand the functioning of the human microbiome, especially the TMME. The microbiome also includes viruses (especially bacteriophages) and fungi, which also play a significant role in the development and course of cancer (75, 76). Moreover, it is very important to expand the field of research to other less common types of cancer. The current literature focuses mainly on the most common cancers, excluding all others.

We are becoming convinced that cancers are highly diversified and internally heterogeneous and have too many survival mechanisms for one simple substance to fight them effectively. Bacteria are microorganisms that existed on Earth approximately 1 billion years before the emergence of the first eukaryotic cells, and their diversity and ability to adapt to all environments aroused admiration among scientists. The tropism of certain bacteria toward the TME and their natural cytotoxic and immunomodulatory properties combined with easy genetic manipulation allows us to conclude that bacteriotherapy may become an anticancer therapy in the future. Unfortunately, the results of clinical trials are still unsatisfactory. Therefore, in addition to attempting to develop new therapies, understanding the complexity and interactions within the TME and attempting to translate this knowledge into practical application are key issues; thus, it is possible to save the lives of millions of human beings worldwide.

**Table 1:** Examples of potentially protective bacterial components of nosopharyngeal, skin and intestinal microbiome in selected cancer types (16-24)

Cancer type	Potentially protective bacteria and their localization
Cutaneous T-cell lymphoma (CTCL)	*Staphylococcus capitis, S. epidermidis, S. warneri, Acinetobacter, Anaerococcus *(skin)
Colorectal cancer (CRC)	*Blautia* sp. SC05B48, *Colinsella aerofaciens*, *Lachnospiraceae* bacterium Choco 86,* Enterococcus hirae*, *Blautia* sp. YL58
Gastric cancer (GC)	*Haemophilus* sp., *Neisseria* sp. (oral cavity)
Non-small cell lung cancer (NSCLC)	*Akkermansia municiphila*, *Rikenellaceae*,* Bacteroides* spp., *Peptostreptococcaceae*, *Mogibacteriaceae*, *Clostridium spp*. (intestines)
Nosopharyngeal cancer (NPC)	*Pseudomonas* sp., *Acinetobacter* sp
Various cancer types	*Bifidobacterium* spp., *Lactobacillus* spp. (intestines)

* *P. aeruginosa* way into the tumor may be different, **Cells and (bio)chemical compounds are not in scale

TAM: Tumor-Associated Macrophages, PQS: *Pseudomonas Quinolone* Signal, IL: interleukine, TGF: Tumor Growth Factor, OdDHL: N-3-oxo-dodecanoyl-L-homoserine lactone, DCs: dendritic cells, TME: tumor microenvironment (38-44)

1) *Salmonella Typhimurium* as a carrier of proapoptotic and immunostimulatory proteins; 2) *Synechococcus* sp. in photodynamic therapy; 3) Self-attenuating *Listeria monocytogenes* vaccine

TAM: Tumor-associated Macrophages, ROS: Reactive Oxygen Species, ActA: Actine Assembly-inducing protein, LoxP: Locus of X-over P1 (52, 53, 57, 66, 67)

*Cells and biochemical compounds are not in scale

## Conclusion

The composition of intestinal, skin, and lung microbiota differs significantly between healthy and cancer patients, influencing tumor development and survival. Bacteriome within the TME participates in diversified course of the disease and treatment. *A. municiphila, P. aeruginosa, *and *Bifidobacterium *sp. are examples of TME bacteria having anti-cancer characteristics. Advances in bacteriotherapy development are possible with genetic engineering and synthetic biology. Advanced human microbiome analyses, including tumor bacteriome, are crucial to understanding complex interactions between microorganisms, cancer, and immune cells. This knowledge may be used for diagnostic and treatment possibilities in the future. Microbiome modulation and bacteriotherapy are giving fascinating results *in vitro* and *in vivo* and should be of particular interest in the cancer research field. What’s crucial is that interest in this research field is continuously growing, giving a lot of hope for the future.

## Declaration

We have not used any AI tools or technologies to prepare this manuscript.
